# Supplementary material for: A fault-intrusion-tolerant system and deadline-aware algorithm for scheduling scientific workflow in the cloud
Source: PeerJ Comput Sci. 2021 Nov 2;7:e747. doi: 10.7717/peerj-cs.747 (PMC8576554; doi:10.7717/peerj-cs.747)
Supplement: Supplemental Information 6 [file peerj-cs-07-747-s006.zip › Myproject1.1/docs/org/cloudbus/cloudsim/CloudletScheduler.html]

CloudletScheduler


JavaScript is disabled on your browser.


- Overview
- Package
- Class
- Use
- Tree
- Deprecated
- Index
- Help

- Prev Class
- Next Class

- Frames
- No Frames

- All Classes

- Summary:
- Nested |
- Field |
- Constr |
- Method

- Detail:
- Field |
- Constr |
- Method


org.cloudbus.cloudsim

## Class CloudletScheduler

- java.lang.Object
- - org.cloudbus.cloudsim.CloudletScheduler

- Direct Known Subclasses:
  :   CloudletSchedulerSpaceShared, CloudletSchedulerTimeShared, NetworkCloudletSpaceSharedScheduler

  ---

    

  ```
  public abstract class CloudletScheduler
  extends java.lang.Object
  ```

  CloudletScheduler is an abstract class that represents the policy of scheduling performed by a
  virtual machine. So, classes extending this must execute Cloudlets. Also, the interface for
  cloudlet management is also implemented in this class.

  Since:
  :   CloudSim Toolkit 1.0

- - ### Constructor Summary

    Constructors

    Constructor and Description

    `CloudletScheduler()`

    Creates a new CloudletScheduler object.
  - ### Method Summary

    Methods

    | Modifier and Type | Method and Description |
    | --- | --- |
    | `abstract Cloudlet` | `cloudletCancel(int clId)` Cancels execution of a cloudlet. |
    | `abstract void` | `cloudletFinish(ResCloudlet rcl)` Processes a finished cloudlet. |
    | `abstract boolean` | `cloudletPause(int clId)` Pauses execution of a cloudlet. |
    | `abstract double` | `cloudletResume(int clId)` Resumes execution of a paused cloudlet. |
    | `abstract double` | `cloudletSubmit(Cloudlet gl)` Receives an cloudlet to be executed in the VM managed by this scheduler. |
    | `abstract double` | `cloudletSubmit(Cloudlet gl, double fileTransferTime)` Receives an cloudlet to be executed in the VM managed by this scheduler. |
    | `abstract int` | `getCloudletStatus(int clId)` Gets the status of a cloudlet. |
    | `java.util.List<java.lang.Double>` | `getCurrentMipsShare()` Gets the current mips share. |
    | `abstract java.util.List<java.lang.Double>` | `getCurrentRequestedMips()` Gets the current requested mips. |
    | `abstract double` | `getCurrentRequestedUtilizationOfBw()` Gets the current requested bw. |
    | `abstract double` | `getCurrentRequestedUtilizationOfRam()` Gets the current requested ram. |
    | `abstract Cloudlet` | `getNextFinishedCloudlet()` Returns the next cloudlet in the finished list, $null if this list is empty. |
    | `double` | `getPreviousTime()` Gets the previous time. |
    | `abstract double` | `getTotalCurrentAllocatedMipsForCloudlet(ResCloudlet rcl, double time)` Gets the total current allocated mips for cloudlet. |
    | `abstract double` | `getTotalCurrentAvailableMipsForCloudlet(ResCloudlet rcl, java.util.List<java.lang.Double> mipsShare)` Gets the total current mips for the Cloudlet. |
    | `abstract double` | `getTotalCurrentRequestedMipsForCloudlet(ResCloudlet rcl, double time)` Gets the total current requested mips for cloudlet. |
    | `abstract double` | `getTotalUtilizationOfCpu(double time)` Get utilization created by all cloudlets. |
    | `abstract boolean` | `isFinishedCloudlets()` Informs about completion of some cloudlet in the VM managed by this scheduler. |
    | `abstract Cloudlet` | `migrateCloudlet()` Returns one cloudlet to migrate to another vm. |
    | `abstract int` | `runningCloudlets()` Returns the number of cloudlets runnning in the virtual machine. |
    | `protected void` | `setCurrentMipsShare(java.util.List<java.lang.Double> currentMipsShare)` Sets the current mips share. |
    | `protected void` | `setPreviousTime(double previousTime)` Sets the previous time. |
    | `abstract double` | `updateVmProcessing(double currentTime, java.util.List<java.lang.Double> mipsShare)` Updates the processing of cloudlets running under management of this scheduler. |

    - ### Methods inherited from class java.lang.Object

      `clone, equals, finalize, getClass, hashCode, notify, notifyAll, toString, wait, wait, wait`

- - ### Constructor Detail


    - #### CloudletScheduler

      ```
      public CloudletScheduler()
      ```

      Creates a new CloudletScheduler object. This method must be invoked before starting the
      actual simulation.
  - ### Method Detail


    - #### updateVmProcessing

      ```
      public abstract double updateVmProcessing(double currentTime,
                              java.util.List<java.lang.Double> mipsShare)
      ```

      Updates the processing of cloudlets running under management of this scheduler.

      Parameters:
      :   `currentTime` - current simulation time
      :   `mipsShare` - array with MIPS share of each processor available to the scheduler

      Returns:
      :   time predicted completion time of the earliest finishing cloudlet, or 0 if there is no
          next events


    - #### cloudletSubmit

      ```
      public abstract double cloudletSubmit(Cloudlet gl,
                          double fileTransferTime)
      ```

      Receives an cloudlet to be executed in the VM managed by this scheduler.

      Parameters:
      :   `gl` - the submited cloudlet
      :   `fileTransferTime` - time required to move the required files from the SAN to the VM

      Returns:
      :   expected finish time of this cloudlet, or 0 if it is in a waiting queue


    - #### cloudletSubmit

      ```
      public abstract double cloudletSubmit(Cloudlet gl)
      ```

      Receives an cloudlet to be executed in the VM managed by this scheduler.

      Parameters:
      :   `gl` - the submited cloudlet

      Returns:
      :   expected finish time of this cloudlet, or 0 if it is in a waiting queue


    - #### cloudletCancel

      ```
      public abstract Cloudlet cloudletCancel(int clId)
      ```

      Cancels execution of a cloudlet.

      Parameters:
      :   `clId` - ID of the cloudlet being cancealed

      Returns:
      :   the canceled cloudlet, $null if not found


    - #### cloudletPause

      ```
      public abstract boolean cloudletPause(int clId)
      ```

      Pauses execution of a cloudlet.

      Parameters:
      :   `clId` - ID of the cloudlet being paused

      Returns:
      :   $true if cloudlet paused, $false otherwise


    - #### cloudletResume

      ```
      public abstract double cloudletResume(int clId)
      ```

      Resumes execution of a paused cloudlet.

      Parameters:
      :   `clId` - ID of the cloudlet being resumed

      Returns:
      :   expected finish time of the cloudlet, 0.0 if queued


    - #### cloudletFinish

      ```
      public abstract void cloudletFinish(ResCloudlet rcl)
      ```

      Processes a finished cloudlet.

      Parameters:
      :   `rcl` - finished cloudlet


    - #### getCloudletStatus

      ```
      public abstract int getCloudletStatus(int clId)
      ```

      Gets the status of a cloudlet.

      Parameters:
      :   `clId` - ID of the cloudlet

      Returns:
      :   status of the cloudlet, -1 if cloudlet not found


    - #### isFinishedCloudlets

      ```
      public abstract boolean isFinishedCloudlets()
      ```

      Informs about completion of some cloudlet in the VM managed by this scheduler.

      Returns:
      :   $true if there is at least one finished cloudlet; $false otherwise


    - #### getNextFinishedCloudlet

      ```
      public abstract Cloudlet getNextFinishedCloudlet()
      ```

      Returns the next cloudlet in the finished list, $null if this list is empty.

      Returns:
      :   a finished cloudlet


    - #### runningCloudlets

      ```
      public abstract int runningCloudlets()
      ```

      Returns the number of cloudlets runnning in the virtual machine.

      Returns:
      :   number of cloudlets runnning


    - #### migrateCloudlet

      ```
      public abstract Cloudlet migrateCloudlet()
      ```

      Returns one cloudlet to migrate to another vm.

      Returns:
      :   one running cloudlet


    - #### getTotalUtilizationOfCpu

      ```
      public abstract double getTotalUtilizationOfCpu(double time)
      ```

      Get utilization created by all cloudlets.

      Parameters:
      :   `time` - the time

      Returns:
      :   total utilization


    - #### getCurrentRequestedMips

      ```
      public abstract java.util.List<java.lang.Double> getCurrentRequestedMips()
      ```

      Gets the current requested mips.

      Returns:
      :   the current mips


    - #### getTotalCurrentAvailableMipsForCloudlet

      ```
      public abstract double getTotalCurrentAvailableMipsForCloudlet(ResCloudlet rcl,
                                                   java.util.List<java.lang.Double> mipsShare)
      ```

      Gets the total current mips for the Cloudlet.

      Parameters:
      :   `rcl` - the rcl
      :   `mipsShare` - the mips share

      Returns:
      :   the total current mips


    - #### getTotalCurrentRequestedMipsForCloudlet

      ```
      public abstract double getTotalCurrentRequestedMipsForCloudlet(ResCloudlet rcl,
                                                   double time)
      ```

      Gets the total current requested mips for cloudlet.

      Parameters:
      :   `rcl` - the rcl
      :   `time` - the time

      Returns:
      :   the total current requested mips for cloudlet


    - #### getTotalCurrentAllocatedMipsForCloudlet

      ```
      public abstract double getTotalCurrentAllocatedMipsForCloudlet(ResCloudlet rcl,
                                                   double time)
      ```

      Gets the total current allocated mips for cloudlet.

      Parameters:
      :   `rcl` - the rcl
      :   `time` - the time

      Returns:
      :   the total current allocated mips for cloudlet


    - #### getCurrentRequestedUtilizationOfRam

      ```
      public abstract double getCurrentRequestedUtilizationOfRam()
      ```

      Gets the current requested ram.

      Returns:
      :   the current requested ram


    - #### getCurrentRequestedUtilizationOfBw

      ```
      public abstract double getCurrentRequestedUtilizationOfBw()
      ```

      Gets the current requested bw.

      Returns:
      :   the current requested bw


    - #### getPreviousTime

      ```
      public double getPreviousTime()
      ```

      Gets the previous time.

      Returns:
      :   the previous time


    - #### setPreviousTime

      ```
      protected void setPreviousTime(double previousTime)
      ```

      Sets the previous time.

      Parameters:
      :   `previousTime` - the new previous time


    - #### setCurrentMipsShare

      ```
      protected void setCurrentMipsShare(java.util.List<java.lang.Double> currentMipsShare)
      ```

      Sets the current mips share.

      Parameters:
      :   `currentMipsShare` - the new current mips share


    - #### getCurrentMipsShare

      ```
      public java.util.List<java.lang.Double> getCurrentMipsShare()
      ```

      Gets the current mips share.

      Returns:
      :   the current mips share


- Overview
- Package
- Class
- Use
- Tree
- Deprecated
- Index
- Help

- Prev Class
- Next Class

- Frames
- No Frames

- All Classes

- Summary:
- Nested |
- Field |
- Constr |
- Method

- Detail:
- Field |
- Constr |
- Method
